# *Scleraxis* is required for maturation of tissue domains for proper integration of the musculoskeletal system

**DOI:** 10.1038/srep45010

**Published:** 2017-03-22

**Authors:** Yuki Yoshimoto, Aki Takimoto, Hitomi Watanabe, Yuji Hiraki, Gen Kondoh, Chisa Shukunami

**Affiliations:** 1 Department of Molecular Biology and Biochemistry, Division of Basic Life Sciences, Institute of Biomedical and Health Sciences, Hiroshima University, Hiroshima, 734-8553, Japan; 2 Laboratory of Cellular Differentiation, Institute for Frontier Life and Medical Sciences, Kyoto University, Kyoto, 606-8507, Japan; 3 Laboratory of Animal Experiment for Regeneration, Institute for Frontier Life and Medical Sciences, Kyoto University, Kyoto, 606-8507, Japan

## Abstract

Scleraxis (Scx) is a basic helix-loop-helix transcription factor that is expressed persistently in tendons/ligaments, but transiently in entheseal cartilage. In this study, we generated a novel *Scx*^*Cre*^ knock-in (KI) allele, by in-frame replacement of most of *Scx* exon 1 with *Cre recombinase (Cre*), to drive *Cre* expression using *Scx* promoter and to inactivate the endogenous *Scx*. Reflecting the intensity and duration of endogenous expression, Cre-mediated excision occurs in tendinous and ligamentous tissues persistently expressing *Scx*. Expression of tenomodulin, a marker of mature tenocytes and ligamentocytes, was almost absent in tendons and ligaments of *Scx*^*Cre*/*Cre*^ KI mice lacking *Scx* to indicate defective maturation. In homozygotes, the transiently *Scx*-expressing entheseal regions such as the rib cage, patella cartilage, and calcaneus were small and defective and cartilaginous tuberosity was missing. Decreased Sox9 expression and phosphorylation of Smad1/5 and Smad3 were also observed in the developing entheseal cartilage, patella, and deltoid tuberosity of *Scx*^*Cre*/*Cre*^ KI mice. These results highlighted the functional importance of both transient and persistent expression domains of *Scx* for proper integration of the musculoskeletal components.

During early stages of musculoskeletal development, scleraxis (Scx), a basic helix-loop-helix transcription factor, is expressed in the tendon/ligament cell population and the subpopulation of chondrogenic cells that contribute to establishment of chondro-tendinous/ligamentous junctions^1^,^2^,^3^,^4^. *Scx* expression occurs only transiently in the chondrogenic lineage because it is rapidly downregulated during the early stages of chondrogenesis^3^. In contrast, *Scx* is persistently expressed in the tendon/ligament cell lineage, which also includes the diaphragm and cordae tendinae of the heart, during differentiation and maturation^5^,^6^,^7^,^8^. Persistent expression of *Scx*, in the developing tendons and ligaments *in vivo*, was also visualised in *ScxGFP* reporter transgenic mice that faithfully recapitulate endogenous *Scx*-expressing domains under the control of the promoter/enhancer of mouse *Scx*^3^,^9^. In *Scx*^−/−^ embryos, force-transmitting and intermuscular tendons are reported to be severely affected and are hypoplastic^7^. However, so far, other *Scx*-expressing domains have not been explored in detail yet.

Tenomodulin (Tnmd) is an excellent cell surface marker to evaluate differentiation and maturation of dense connective tissues, including tendons and ligaments^10^,^11^,^12^,^13^. Scx positively regulates the expression of *type I collagen (Col1*) and *Tnmd* in tenocytes^6^,^7^,^14^. Transcriptome analysis of mouse embryonic limb tendons also revealed that *Tnmd* is the second most differentially expressed gene of the top 100 genes differentially expressed between E11.5 and E14.5^15^. Studies on *Tnmd* null mice demonstrated that *Tnmd* is necessary for tenocyte proliferation and tendon maturation^16^. Cellular adhesion in the periodontal ligament is decreased on loss of *Tnmd* and enhanced on *Tnmd* overexpression^17^. In a rat rotator cuff healing model, fibroblast growth factor-2 enhanced tendon-to-bone healing, in which *Tnmd* expression was associated with formation of aligned collagen fibres in the repair tissue^18^. Loss of *Tnmd* results in reduced self-renewal and augmented senescence of tendon stem/progenitor cells^19^. Furthermore, forced expression of *Scx* resulted in the conversion of human bone marrow-derived mesenchymal stem cells into tendon progenitor cells that differentiate into *Tnmd*-expressing cells^20^. Additionally in tenocytes, *Tnmd* expression is markedly upregulated by retroviral overexpression of *Scx*^6^,^21^.

In this study, we report a novel *Scx*^*Cre*^ knock-in (KI) mouse line that was generated by in-frame replacement of most of the exon 1 on mouse *Scx* locus with *Cre recombinase (Cre*), to inactivate endogenous *Scx* and to drive *Cre* expression using the endogenous *Scx* promoter. In this established *Scx*^*Cre*^ KI line, *Cre* expression was detected using *Rosa-CAG-LSL-tdTomato (Rosa-tdTomato*) reporter mice expressing tandem dimer Tomato (tdTomato) by Cre-mediated recombination. A number of tdTomato-expressing cells of *Scx*^*Cre*/+^; *Rosa-tdTomato* or *Scx*^*Cre*/*Cre*^; *Rosa-tdTomato* mice were observed in tendons, ligaments, annulus fibrosus of intervertebral discs, and patella cartilage. Loss of *Scx* resulted in an almost absent expression of Tnmd in tendons, ligaments, and annulus fibrosus of intervertebral discs. Cartilaginous elements transiently expressing *Scx* were defective in *Scx*^*Cre*/*Cre*^ KI mice lacking *Scx*. Sox9 expression and phosphorylation of Smad1/5 and Smad 3 were markedly decreased in these *Scx*^+^ cartilaginous elements. Our results clearly demonstrated the functional importance of *Scx* in maturation of tissue domains that express *Scx* both persistently and transiently during development.

## Results

### Establishment of *Scx* ^*Cre*^ KI mice

**Figure 1:**
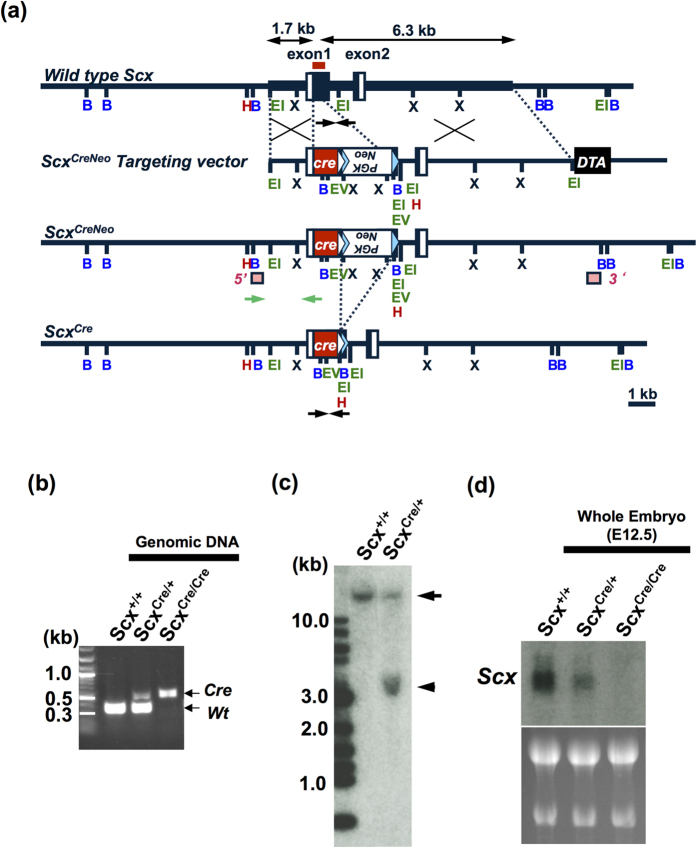
Targeting strategy to generate *Scx*^*Cre*^ KI mice. (**a**) The mouse *scleraxis (Scx*) gene consists of exon 1 and 2. Black boxes represent the coding regions of *Scx* gene, whereas open boxes represent 5′ and 3′ untranslated regions. The coding region of *Cre recombinase (Cre*) is shown as red boxes. The neomycin-resistance (*Neo*) cassette with phosphoglycerate kinase I (*PGK*) promoter (white open boxes) was used for positive selection of embryonic stem (ES) cells. Diphtheria toxin (DTA) was used for negative selection. White and light blue triangles indicate *loxP* and *FRT* sites. Restriction enzyme sites for *Bam*HI (B), *Hin*dIII (H), *Eco*RI (EI), *Eco*RV (EV), and *Xba*I (X) are shown. Arrows on wild type allele indicate the 5′ (1.7 kb) and 3′ (6.3 kb) homology arms. To generate *Scx*^*Cre*^ allele, the *Neo* cassette was removed by crossing *Scx*^*CreNeo*^ mice with flippase-expressing *FLPe* mice. Black arrows under wild type allele and *Scx*^*Cre*^ allele are genotyping primers for detection of wild type and *Scx*^*Cre*^ alleles, respectively. Green arrows under *Scx*^*CreNeo*^ allele are genotyping primers for ES screening. The 5′ and 3′ probes for Southern blotting are shown as pink boxes. Red line on wild type allele shows the region used for RNA probes in northern blot analysis. Scale bar represents 1 kb. (**b**) Tail tip DNA PCR genotyping was performed with primers shown as black arrows in (**a**). The targeted *Scx*^*Cre*^ allele (0.5 kb) and wild type (Wt) allele (0.3 kb) are distinguished. (**c**) Southern blot analysis of mouse genomic DNA isolated from wild type (*Scx*^+/+^) and *Scx*^*Cre*/+^ KI mice. An arrowhead and an arrow indicate the *Scx*^*Cre*^ allele (3.5 kb) and wild type allele (19.1 kb), respectively. Hybridised bands on the far left show DNA size markers. (**d**) Detection of *Scx* transcripts in whole embryos at E12.5. *Scx* expression in *Scx*^+/+^, *Scx*^*Cre*/+^, and *Scx*^*Cre*/*Cre*^ KI mice were examined by northern blot analysis. Fifteen micrograms of the total RNA was loaded into each well and equal loading was verified by ethidium bromide staining.

On mouse chromosome 15, *Scx* gene, consisting of two exons, is located within the third intron of *block of proliferation 1 (Bop1*), which is transcribed in the opposite orientation^9^,^22^. The targeting strategy placed *Cre* expression under the control of *Scx* promoter and led to the inactivation of *Scx* allele by in-frame replacement of most of its exon 1, which encodes for most of the coding region, including a b-HLH region. The linearised *Scx*^*CreNeo*^ targeting vector (Fig. 1a) was electroporated into KY1.1 embryonic stem (ES) cell line (C57BL/6J × 129S6/SvEvTac)^23^. ES cell clone with the correct targeting event was microinjected into blastocysts to obtain chimeric mice. We generated heterozygous mice by crossing these chimeras with C57BL/6 wild type mice and confirmed the successful germ line transmission of *Scx*^*CreNeo*^ targeted allele. Heterozygous *Scx*^*CreNeo*/+^ KI mice were viable, whereas homozygous *Scx*^*CreNeo*/*CreNeo*^ KI mice were embryonic lethal. This is consistent with the previous report that conventional *Scx* knockout mice die around embryonic day (E)10.5^24^. To obtain *Scx*^*Cre*/+^ KI mice, we deleted the *flippase (FLP) recombinase target (FRT*)-flanked *neomycin-resistance (Neo*) cassette by mating *Scx*^*CreNeo*/+^ KI mice with mice expressing *FLP recombinase (FLPe*)^25^. The correctly targeted event of *Scx*^*Cre*/+^ KI mice was confirmed by PCR of tail tip DNA and Southern blot analysis (Fig. 1b,c). Expression of *Scx* was not detected in a *Scx*^*Cre*/*Cre*^ KI mouse embryo at E12.5 (Fig. 1d). Thus, we successfully disrupted endogenous *Scx* by inserting a *Cre* in-frame into the ATG start site of mouse *Scx* gene.

### Cre-mediated recombination in *Scx* ^*Cre*^ *KI*; *Rosa-tdTomato*

**Figure 2:**
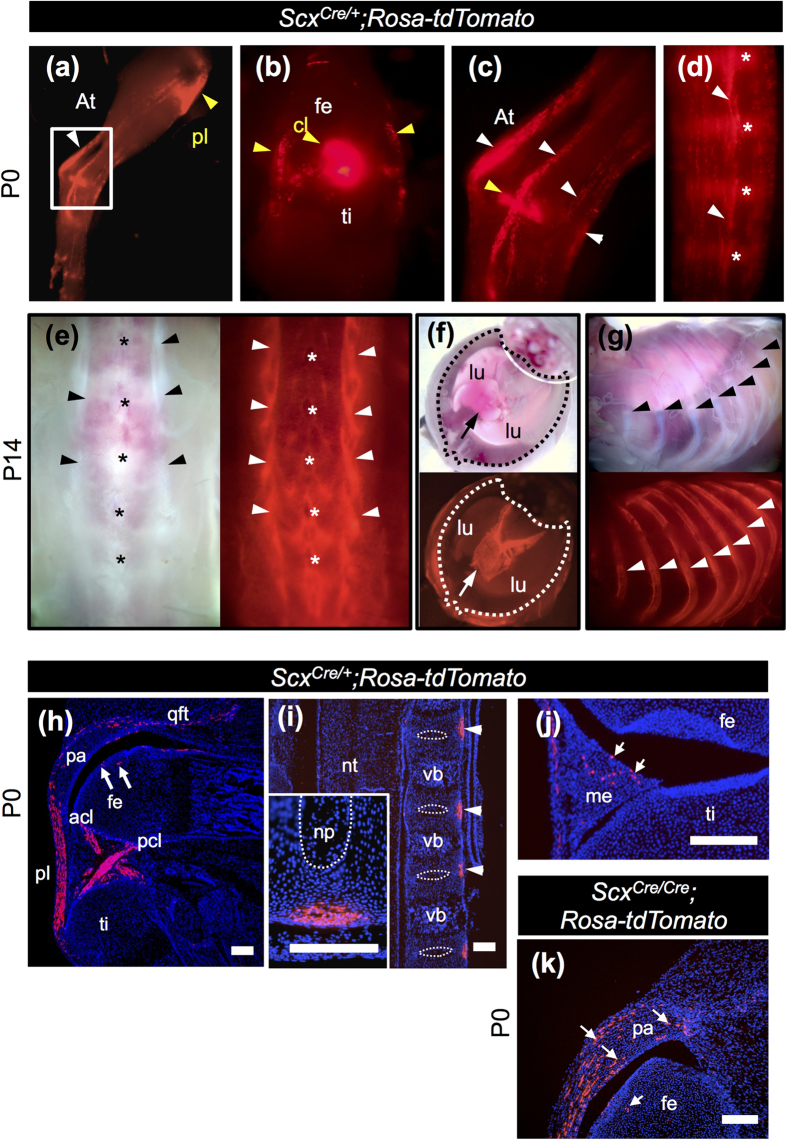
Cre-mediated recombination in tendons, ligaments, and diaphragm of *Scx*^*Cre*/+^; *Rosa-tdTomato* mice. (**a**–**g**) *Cre*-mediated tdTomato expression in P0 (**a**–**d**) and P14 (**e**–**g**) *Scx*^*Cre*/+^; *Rosa-tdTomato* mice. Bright fluorescence was detected in leg tendons (**a**–**c**), tail tendons (**d**), back tendons (**e**), diaphragm (**f**), and costal tendons (**g**). (**a**–**c**) Lateral (**a**,**c**) and frontal (**b**) views of skinned whole mount leg of *Scx*^*Cre*/+^; *Rosa-tdTomato* mice. White and yellow arrowheads in (**a**–**c**) indicate tendons and ligaments of the hindlimb, respectively. Arrowheads in (**d**) indicate tail tendons. Asterisks in (**d**) indicate intervertebral region. (**e**–**g**) Bright and fluorescent images of dorsal view of lumbar tendons (**e**), ventral view of diaphragm (**f**), and lateral view of costal tendons (**g**) are shown. Arrowheads and asterisks in (**e**) indicate tendons and spinous processes, respectively. Arrows in (**f**) indicate central tendon of diaphragm. Arrowheads in (**g**) indicate ribs. Dotted line in (**f**) encloses the outer boundary of diaphragm. (**h**–**k**) Sagittal sections of knee joint (**h**,**j**,**k**) and vertebral column (**i**) prepared from *Scx*^*Cre*/+^; *Rosa-tdTomato* (**h**–**j**) and *Scx*^*Cre*/*Cre*^; *Rosa-tdTomato* (**k**) neonates. An inset in (**i**) shows magnified image of ventral annulus fibrosus. Arrows in (**h**,**j**,**k**) indicate tdTomato-positive chondrocytes in patella, meniscus, and femur, respectively. acl, anterior cruciate ligament; At, Achilles tendon; cl, cruciate ligament; fe, femur; lu, lung; pa, patella; pcl, posterior cruciate ligament; me, meniscus; np, nucleus pulposus; nt, neural tube; pl, patella ligament; qft, quadriceps femoris tendon; ti, tibia; vb, vertebral body. Scale bars, 200 μm (**h**–**k**); 100 μm (inset in (**i**)).

Cre-mediated recombination was examined by crossing *Scx*^*Cre*/+^ KI mice with *Rosa-tdTomato* reporter mice^26^. Although endogenous expression of *Scx* is detectable around E9.5, Cre-mediated tdTomato expression in *Scx*^*Cre*/+^; *Rosa-tdTomato* was first detected around E16.5 (data not shown). In the hind limbs of *Scx*^*Cre*/+^; *Rosa-tdTomato* neonates, we detected tdTomato expression in the Achilles tendon and in tendons and ligaments associated with knee joint and heel (Fig. 2a–c). Tail tendons of *Scx*^*Cre*/+^; *Rosa-tdTomato* neonates were also positive for tdTomato (Fig. 2d). Moreover, at postnatal day (P)14, more intense expression of tdTomato was observed in body tendons and ligaments (Fig. 2e,f,g). In the trunk of 2-week-old *Scx*^*Cre*/+^; *Rosa-tdTomato* mice, intense tdTomato expression was observed in tendons of longissimus muscle (Fig. 2e), central tendon of diaphragm (Fig. 2f), and costal tendons (Fig. 2g). In the hindlimb of *Scx*^*Cre*/+^; *Rosa-tdTomato* neonates, tdTomato-positive cells were found in patella ligament, anterior and posterior cruciate ligaments, quadriceps femoris tendon, patella cartilage, femur cartilage, and meniscus (Fig. 2h–j). In the intervertebral region of *Scx*^*Cre*/+^; *Rosa-tdTomato* neonates, tdTomato expression was detected in the outer annulus fibrosus on the ventral side (Fig. 2i). In the hindlimb of *Scx*^*Cre*/*Cre*^; *Rosa-tdTomato* neonates, the number of tdTomato-positive cells in patella cartilage increased (Fig. 2k). Mosaic Cre-mediated recombination could have occurred because of heterogeneity in the endogenous *Scx* mRNA levels and time window of *Scx* expression in the *Scx*-expressing cells.

### Tendon deficiency and skeletal abnormalities in *Scx* ^*Cre*/*Cre*^ KI neonates

**Figure 3:**
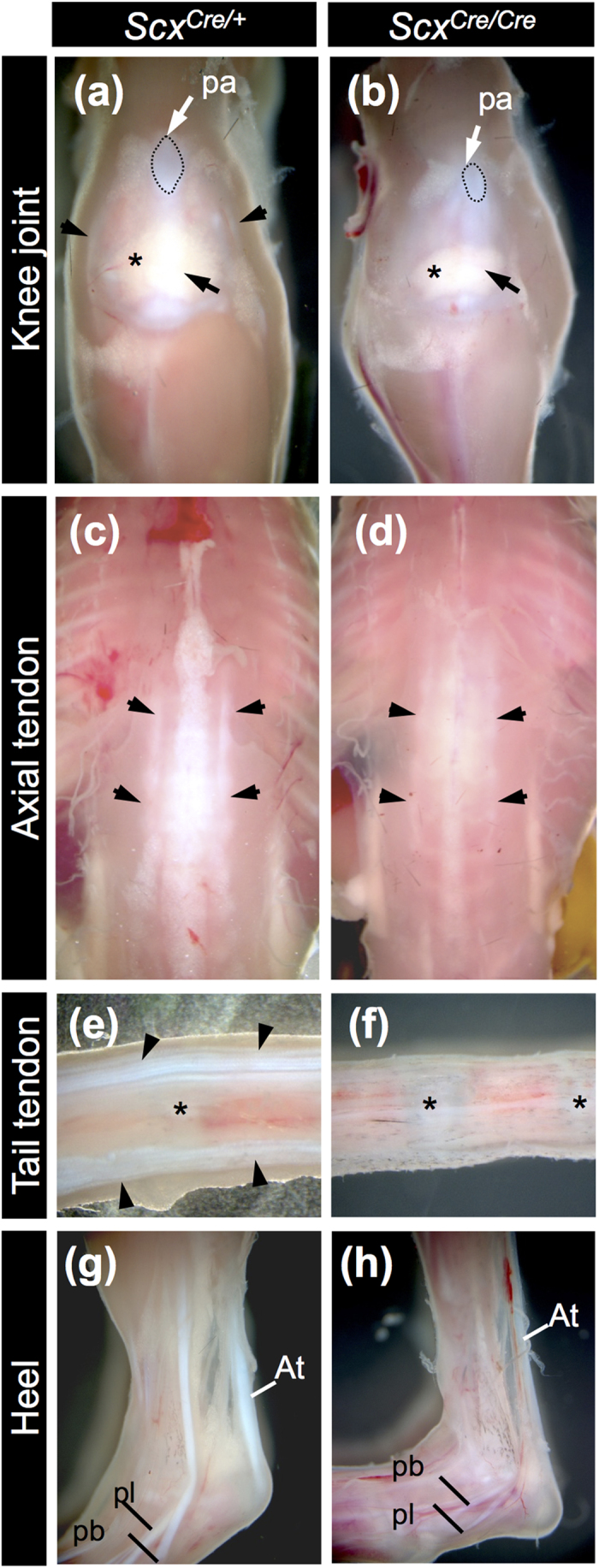
Tendon deficiency in *Scx*^*Cre*/*Cre*^ KI mice. (**a**–**h**) Tendons and ligaments of 3-week-old *Scx*^*Cre*/+^ (**a**,**c**,**e**,**g**) and *Scx*^*Cre*/*Cre*^ (**b**,**d**,**f**,**h**) KI mice. Frontal views of knee (**a**,**b**), dorsal views of back (**c**,**d**), and lateral views of tail (**e**,**f**) and heel (**g**,**h**) are shown. A black and a white arrow in (**a**,**b**) indicate patella ligament and patella, respectively. Arrowheads in (**a**) indicate collateral ligaments. Asterisk in (**a**,**b**) indicates fat pad in the knee joint. Dotted line in (**a**,**b**) encloses the patella. Arrowheads in (**c**,**d**) indicate back tendons. Arrowheads in (**e**) indicate tail tendons. Asterisks in (**e**,**f**) indicate intervertebral region. At, Achilles tendon; pa, patella; pb, peroneus brevis tendon; pl, peroneus longus tendon.

**Figure 4:**
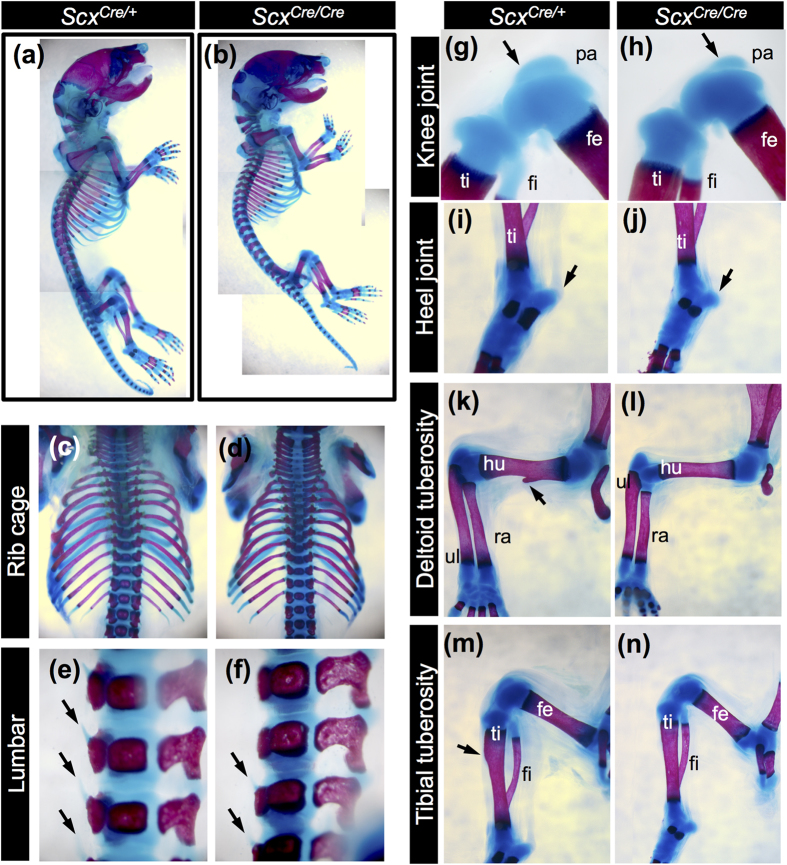
Skeletal abnormalities in *Scx*^*Cre*/*Cre*^ KI mice. (**a**–**n**) Skeletons of *Scx*^*Cre*/+^ (**a**,**c**,**e**,**g**,**i**,**k**,**m**) and *Scx*^*Cre*/*Cre*^ (**b**,**d**,**f**,**h**,**j**,**l**,**n**) KI mice at P0. Lateral (**a**,**b**) and dorsal (**c**,**d**) views of ribs and lateral views of lumbar (**e**,**f**), knee joint (**g**,**h**), heel (**i**,**j**), humerus (**k**,**l**), and lower leg (**m**,**n**) are shown. Arrows in (**e**,**f**) indicate transverse processes of lumbar. Arrows in (**g**,**h**) indicate patella. Arrows in (**k**,**m**) indicate deltoid tuberosity and tibial tuberosity, respectively. fe, femur; fi, fibula; hu, humerus; pa, patella; ra, radius; ti, tibia; ul, ulna.

Heterozygous *Scx*^*Cre*/+^ KI mice were viable, fertile, and displayed no apparent developmental defects (Fig. 3a,c,e,g). Consistent with previous findings^7^, morphological defects in force-transmitting and intermuscular tendons were evident at birth, in *Scx*^*Cre*/*Cre*^ KI neonates (Fig. 3b,d,f,h). The diaphragm of *Scx*^*Cre*/*Cre*^ KI neonates is functional and permits normal breathing. The forelimb autopod of *Scx*^*Cre*/*Cre*^ KI neonates was locked in a dorsal flexure (Fig. 4b), compared to that of *Scx*^*Cre*/+^ KI neonates (Fig. 4a). The deltoid tuberosity (DT) and tibial tuberosity observed in *Scx*^*Cre*/+^ KI neonates (Fig. 4a,k,m) were missing in *Scx*^*Cre*/*Cre*^ KI neonates (Fig. 4b,l,n). The rib cage, transverse process of lumbar, patella cartilage, and entheseal cartilage of calcaneus of *Scx*^*Cre*/*Cre*^ KI neonates were smaller than that of *Scx*^*Cre*/+^ KI neonates (Fig. 4c–j).

### Defective maturation of tendons, ligaments, and annulus fibrosus of intervertebral discs in *Scx* ^*Cre*/*Cre*^ KI neonates

**Figure 5:**
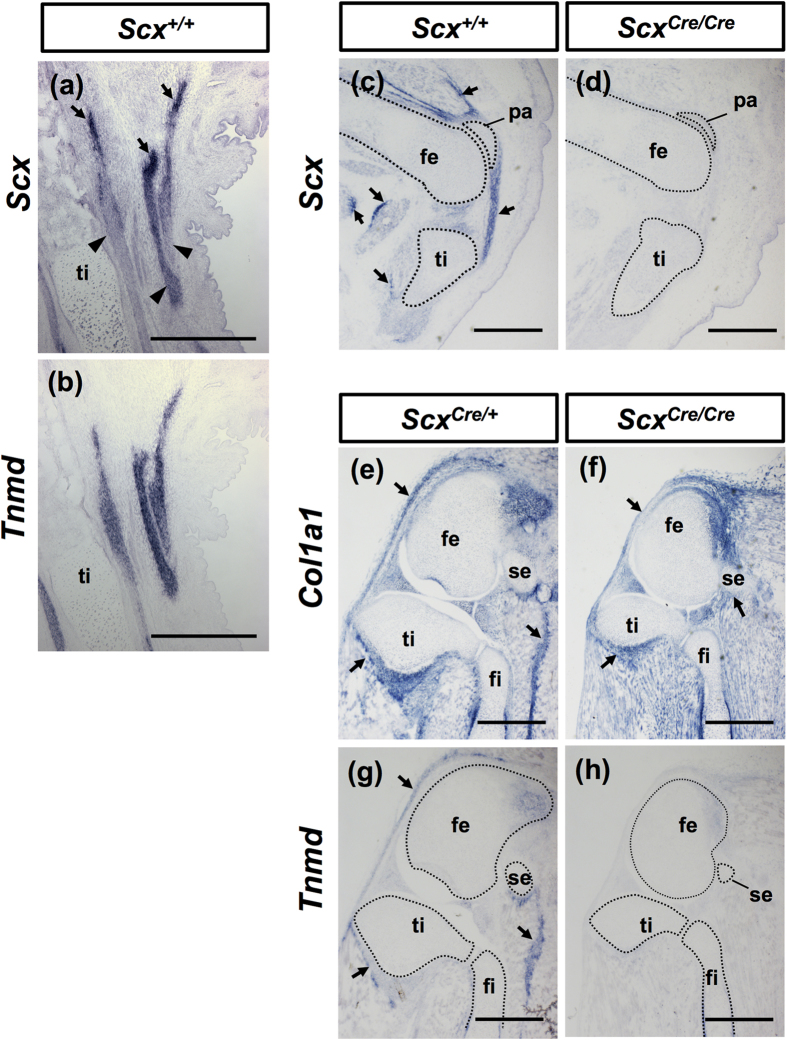
Expression of *Tnmd, Scx*, and *Col1a1* in *Scx*^*Cre*/*Cre*^ KI mice. *In situ* hybridisation of *Scx* (**a**,**c**,**d**), *Tnmd* (**b**,**g**,**h**), and *Col1a1* (**e**,**f**) on sagittal sections of hindlimbs prepared from *Scx*^+/+^ (**a**–**c**), *Scx*^*Cre*/+^ (**e**,**g**), and *Scx*^*Cre*/*Cre*^ (**d**,**f**,**h**) mice at E16.5 (**a**,**b**), E15.5 (**c**,**d**), and P0 (**e**–**h**). Arrows and arrowheads in (**a**) indicate the regions with high and low expression of *Scx*, respectively. Arrows in (**c**,**e**–**g**) indicate *Scx* (**c**), *Col1a1* (**e**,**f**), and *Tnmd* (**g**) positive tendinous region. Dotted lines in (**c**,**d**) and (**g**,**h**) enclose skeletons. fe, femur; fi, fibula; pa, patella; se, sesamoid bone; ti, tibia. Scal bars, 500 μm.

**Figure 6:**
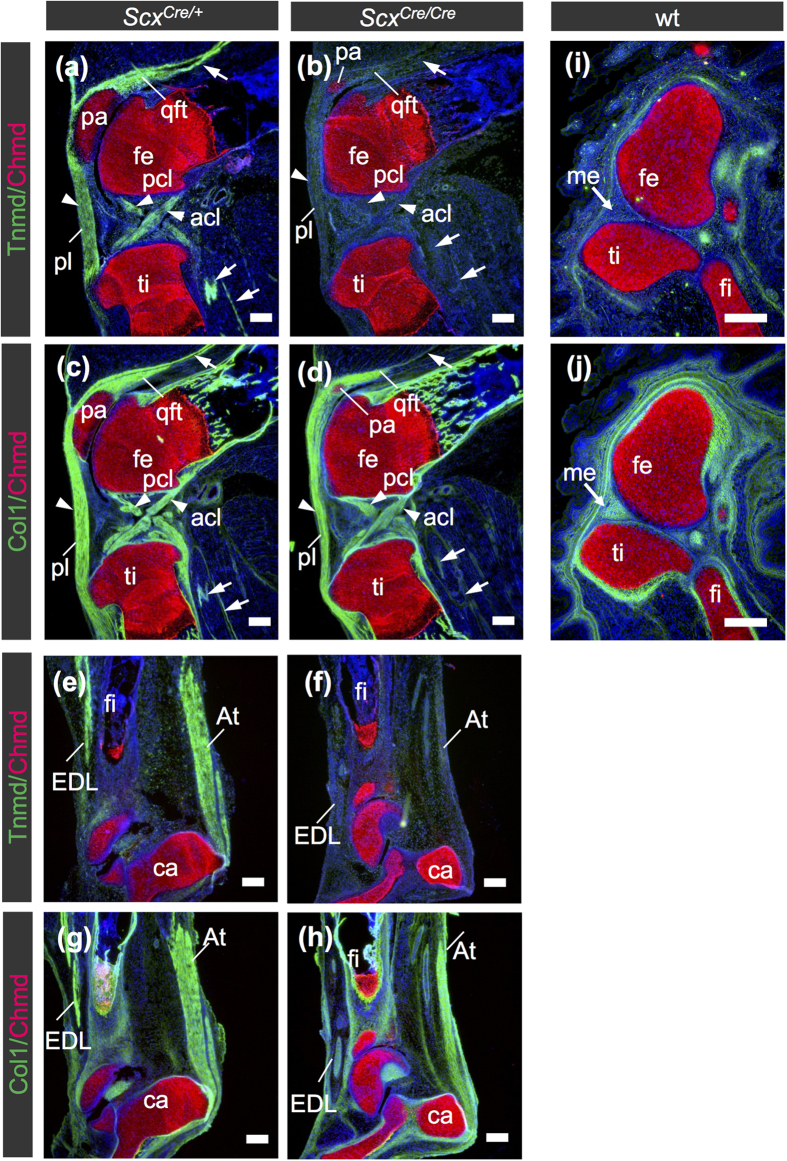
Decreased expression of Tnmd in tendons and ligaments of *Scx*^*Cre*/*Cre*^ KI mice. (**a**–**h**) Double immunostaining of Chmd (red) and Tnmd (green) (**a**,**b**,**e**,**f**,**i**) or Col1 (green) (**c**,**d**,**g**,**h**,**j**) was performed in *Scx*^*Cre*/+^ (**a**,**c**,**e**,**g**) and *Scx*^*Cre*/*Cre*^ (**b**,**d**,**f**,**h**) KI neonates and wild type (wt) embryos at E16.5 (**i**,**j**). Frozen sagittal sections of knee (**a**–**d**,**i**,**j**) and heel (**e**–**h**) are shown. Tnmd (**a**,**e**) and Col1 (**c**,**g**) were detected in tendons and ligaments of *Scx*^*Cre*/+^, whereas Col1-positive tendons and ligaments of *Scx*^*Cre*/*Cre*^ (**d**,**h**) were negative for Tnmd (**b**,**f**). Arrows and arrowheads in (**a**–**d**) indicate tendons and ligaments respectively, whereas arrows in (**i**,**j**) indicate menisci. acl, anterior cruciate ligament; At, Achilles tendon; ca, calcaneus; EDL, extensor digitorum longus tendon; fe, femur; fi, fibula; me, meniscus; pa, patella; pcl, posterior cruciate ligament; pl, patella ligament; qft, quadriceps femoris tendon; ti, tibia. Scale bars, 200 μm.

**Figure 7:**
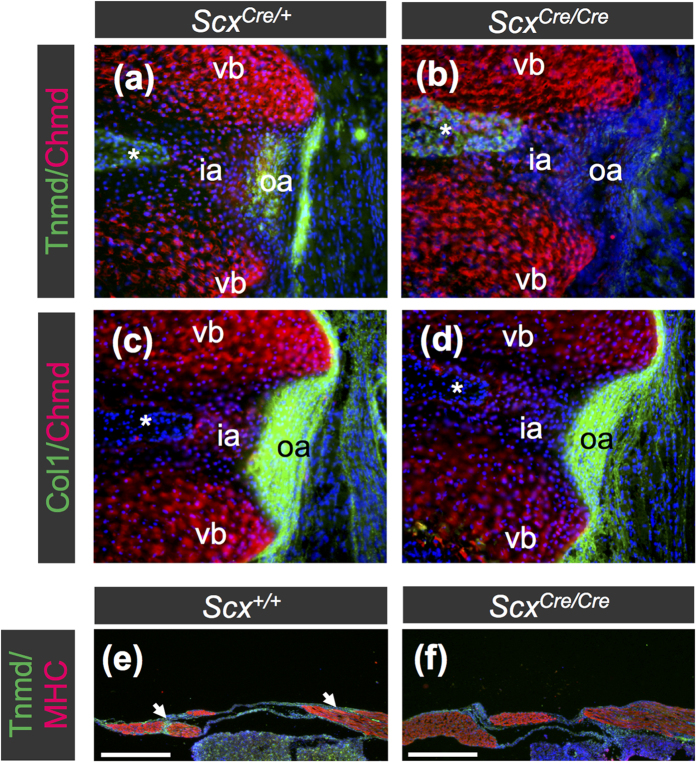
Decreased expression of Tnmd in annulus fibrosus of intervertebral disc and diaphragm of *Scx*^*Cre*/*Cre*^ KI mice. (**a**–**f**) Double immunostaining of Chmd (red) and Tnmd (green) (**a**,**b**) or Col1 (green) (**c**,**d**) was performed in *Scx*^*Cre*/+^ (**a**,**c**) and *Scx*^*Cre*/*Cre*^ (**b**,**d**) KI mice and MHC (red) and Tnmd (green) (**e**,**f**) was performed in *Scx*^+/+^ (e) and *Scx*^*Cre*/*Cre*^ (f) KI mice at P0. Frozen frontal sections of vertebral column (**a**–**d**) and sagittal sections of diaphragm (**e**,**f**) are shown. Tnmd (**a**) and Col1 (**c**) were detected in annulus fibrosus of *Scx*^*Cre*/+^ **KI**, whereas Col1-positive annulus fibrosus of *Scx*^*Cre*/*Cre*^ (**d**) were negative for Tnmd (**b**). Asterisks in (**a**–**d**) indicate nucleus pulposus. Arrows in (**e**) indicate Tnmd-positive tendinous region in diaphragm of *Scx*^+/+^ mouse. ia, inner annulus fibrosus; oa, outer annulus fibrosus; vb, vertebral body. Scale bars, 200 μm.

Tnmd is related to a cartilage-derived angiogenesis inhibitor gene product, chondromodulin (Chmd)^27^, and is a marker of mature tenocytes and ligamentocytes^10^,^11^,^28^. *Tnmd* expression is positively regulated by *Scx*^6^. *Scx* was heterogeneously expressed in the developing leg tendons (Fig. 5a), whereas uniform expression of *Tnmd* was observed (Fig. 5b). Higher levels of *Scx* expression in the tendons were detected near the myotendinous junction (Fig. 5a). We confirmed inactivation of endogenous *Scx* expression in *Scx*^*Cre*/*Cre*^ at E15.5 by *in situ* hybridisation (Fig. 5c,d). As previously reported^7^, in *Scx* null mice, gene expression of *Tnmd* and *type XIV collagen* was not detectable in forelimb tendons. However, during postnatal growth, *Tnmd* expression in other dense connective tissues, such as ligaments and annulus fibrosus of intervertebral discs, has not been evaluated yet. Thus, we investigated localisation of Col1 and Tnmd in tendons and ligaments of *Scx*^*Cre*/+^ and *Scx*^*Cre*/*Cre*^ KI mice, by *in situ* hybridisation (Fig. 5e–h) and double immunostaining (Figs 6 and 7). In the hindlimb of a *Scx*^*Cre*/+^ neonate, tendons and ligaments were positive for Col1 and Tnmd (Fig. 6a,c,e,g). In the knee joint of a homozygous *Scx*^*Cre*/*Cre*^ KI neonate, patella ligament, anterior and posterior cruciate ligaments, and anchoring tendons were positive for Col1, but Tnmd expression was almost absent (Fig. 6b,d). Similarly, in the heel of a homozygous *Scx*^*Cre*/*Cre*^ KI neonate, tendons and ligaments, including the Achilles and extensor digitorum longus tendons, were positive for Col1 but negative for Tnmd (Fig. 6f,h). The meniscus was positive for Col1 but negative for Tnmd at E16.5, despite finding tdTomato positive cells in the meniscus of *Scx*^*Cre*/+^; *Rosa-tdTomato* mice (Figs 2j and 6i,j). The outer annulus fibrosus of intervertebral discs in *Scx*^*Cre*/+^ KI neonates and the tendinous region of the diaphragm in wild type mice were also positive for Col1 and Tnmd (Fig. 7a,c,e), but Tnmd expression was almost absent in that of *Scx*^*Cre*/*Cre*^ KI mice (Fig. 7b,d,f). These results suggest that loss of *Scx* causes defective maturation of tendons, ligaments, and outer annulus fibrosus of intervertebral discs.

### Defective maturation of knee joint of *Scx* ^*Cre*/*Cre*^ KI mice

**Figure 8:**
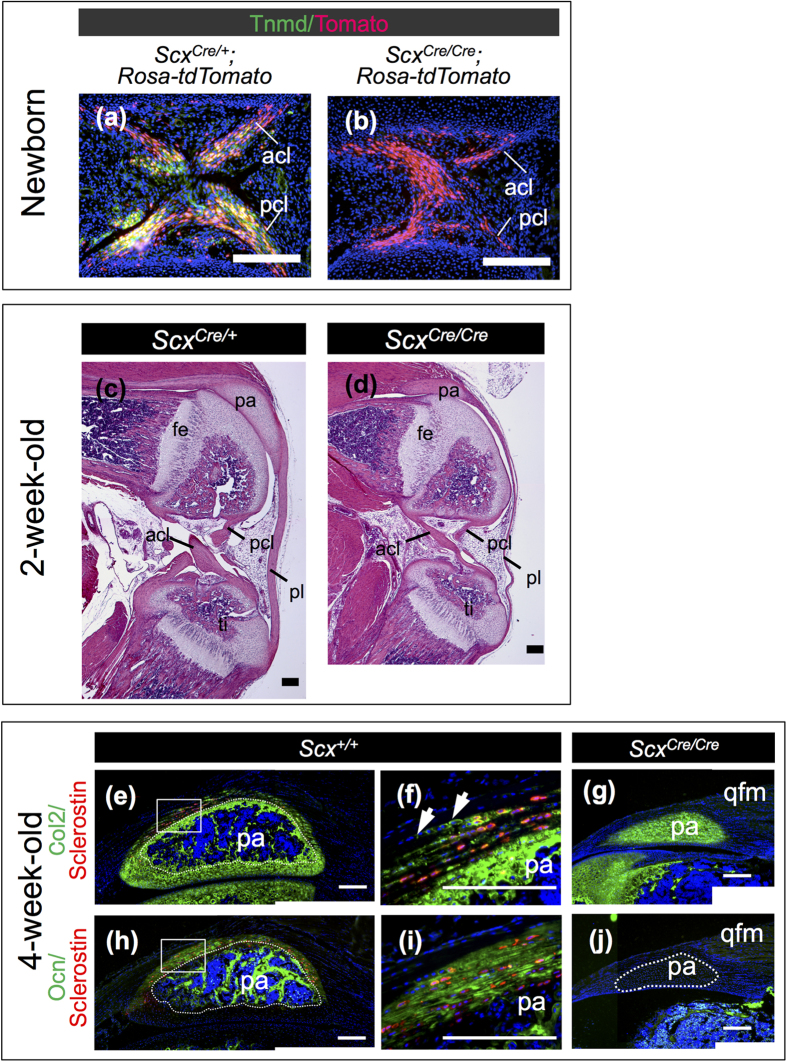
Hypoplastic formation of patella and ligaments in knee joint of *Scx*^*Cre*/*Cre*^ KI mice. (**a**,**b**) Frozen sagittal sections of anterior and posterior cruciate ligaments of *Scx*^*Cre*/+^; *Rosa-tdTomato* (**a**) and *Scx*^*Cre*/*Cre*^; *Rosa-tdTomato* (**b**) are shown. Cre-mediated tdTomato expression was detected as red fluorescence (**a**,**b**). Tnmd-positive cells (green) were visualised by immunostaining using anti-Tnmd antibody. (**c**,**d**) Haematoxylin and eosin staining of sagittal sections of knee joint is shown. Paraffin decalcified specimens of 2-week-old *Scx*^*Cre*/+^ (**c**) and *Scx*^*Cre*/*Cre*^ (**d**) KI mice were sliced. (**e**–**j**) Double immunostaining of sclerostin (red) and Col2 (green) (**e**–**g**) or Ocn (green) (**h**–**j**) was performed on frozen sagittal sections of patella prepared from 4-week-old wild type (**e**,**f**,**h**,**i**) and *Scx*^*Cre*/*Cre*^ (**g**,**j**) KI mice. Boxed regions in (**e**,**h**) are shown at a higher magnification in (**f**,**i**), respectively. Arrows in (**f**) indicate Col2-positive uncalcified fibrochondrocytes. Dotted line in (**e**,**h**) encloses a bone region in the patella. Dotted line in (**j**) encloses patella. acl, anterior cruciate ligament; fe, femur; pa, patella; pcl, posterior cruciate ligament; pl, patella ligament; qfm, quadriceps femoris muscle; ti, tibia. Scale bars, 200 μm.

Tnmd expression, observed in cruciate ligaments of *Scx*^*Cre*/+^; *Rosa-tdTomato* mice, was missing in *Scx*^*Cre*/*Cre*^; *Rosa-tdTomato* mice (Fig. 8a,b). In knee joints of 2-week-old *Scx*^*Cre*/+^ KI mice, tendons and ligaments developed well (Fig. 8c), whereas defective maturation was observed in knee joints of homozygous *Scx*^*Cre*/*Cre*^ KI mice (Fig. 8d). The size of the patella was also significantly small and entheseal cartilage was defective in a *Scx*^*Cre*/*Cre*^ KI mouse (Fig. 8d). To analyse endochondral ossification in the patella, immunostaining using antibodies against type II collagen (Col2), osteocalcin (Ocn), and sclerostin was performed. In a *Scx*^+/+^ mouse, a central part of the patella was vascularised to be replaced by bone stained with Ocn, but Col2-positive staining indicates the presence of cartilaginous matrix (Fig. 8e,f,h,i). At this stage, the patella of a *Scx*^*Cre*/*Cre*^ KI mouse was Col2-positive without any indication of vascularisation and ossification (Fig. 8g,j), suggesting a delay in the endochondral ossification of the patella. In the entheseal region of patella tendon and ligament of a *Scx*^+/+^ mouse, sclerostin-positive cells were observed in Col2- and Ocn-positive calcified fibrocartilage (Fig. 8e,f,h,i). Col2-positive unmineralised fibrocartilage was also present at an adjacent region in patella tendon and ligament of a *Scx*^+/+^ mouse (Fig. 8f). Such a structure was not observed in the *Scx*^*Cre*/*Cre*^ KI mouse. Taken together, *Scx* is also required for maturation of tendons, ligaments, and the patella.

### Decreased Sox9 expression and phosphorylation of Smad1/5 and Smad3 in the developing entheseal cartilage, patella, and deltoid tuberosity of *Scx* ^*Cre*/*Cre*^ KI mice

**Figure 9:**
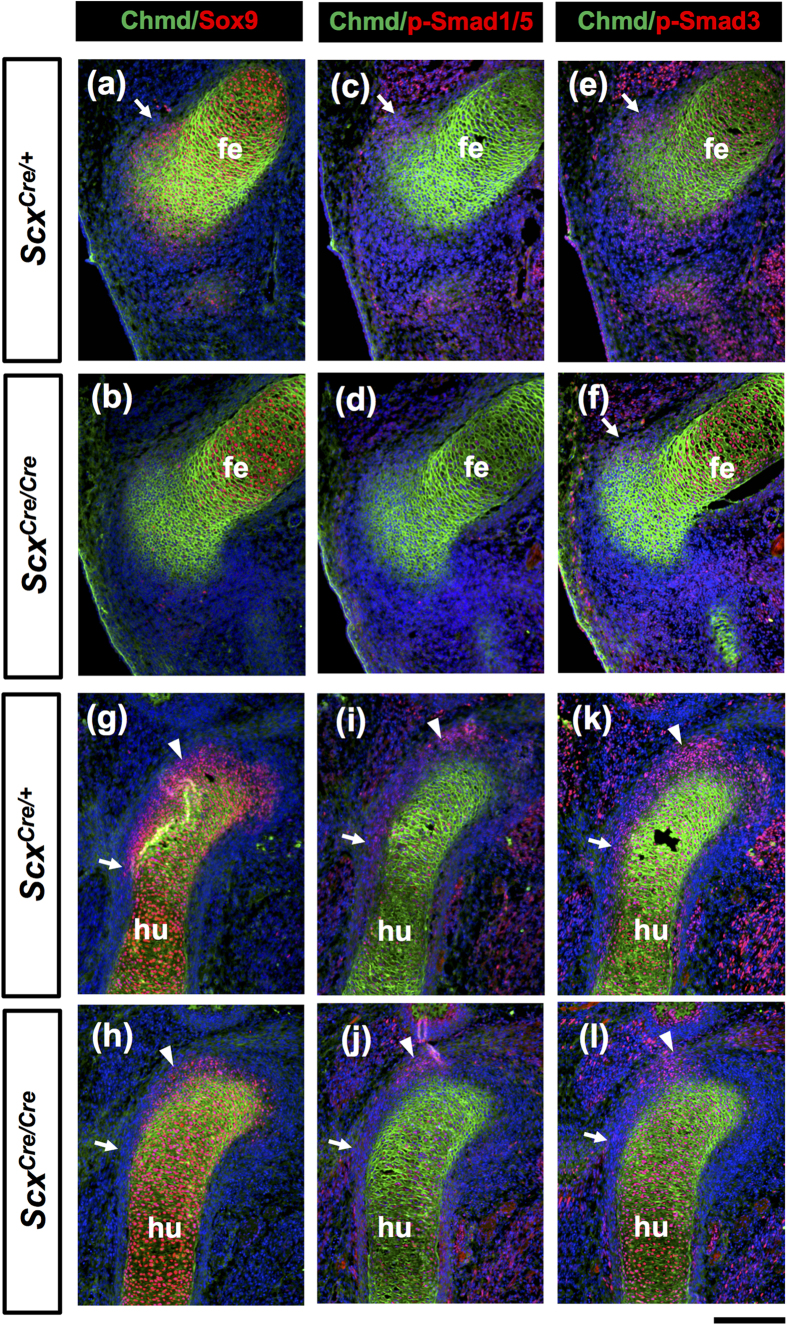
Decreased Sox9 expression and phosphorylation of Smad1/5 and Smad3 in the developing patella and deltoid tuberosity of *Scx*^*Cre*/*Cre*^ KI mouse. Frozen sagittal section of hindlimbs (**a**–**f**) and forelimbs (**g**–**l**) prepared from *Scx*^*Cre*/+^ (**a**,**c**,**e**,**g**,**i**,**k**) and *Scx*^*Cre*/*Cre*^ (**b**,**d**,**f**,**h**,**j**,**l**) KI embryos at E13.5 are shown. (**a**–**l**) Double immunostaining of Chmd (green) and Sox9 (red) (**a**,**b**,**g**,**h**), p-Smad1/5 (red) (**c**,**d**,**i**,**j**), or p-Smad3 (red) (**e**,**f**,**k**,**l**). Arrows in (**a**,**c**,**e**,**f**) indicate developing patella. Arrows and arrowheads in (**g**–**l**) indicate the deltoid tuberosity and the insertion sites of tendon of supraspinous muscle, respectively. fe, femur; hu, humerus. Scale bar, 200 μm.

Entheseal cartilage around the tendon/ligament attachment sites and sesamoid bones such as patella are derived from Scx/Sox9 double positive progenitors^2^,^4^. Blitz *et al*. reported that activation of TGF-β and BMP signaling is required for specification and differentiation of these progenitors, respectively^4^,^29^. Thus, we examined how loss of *Scx* affects expression of Sox9 and activation of these signaling pathways in the developing forelimb and hindlimb of heterozygotes and homozygotes at E13.5. Activation of TGF-β and BMP signaling pathways was monitored by phosphorylation of Smad1/5 and Smad3 that are intracellular downstream mediators^30^. As shown in Fig. 9, mature chondrocytes and progenitor cells of the patella and the deltoid tuberosity were visualized as Chmd positive (green) and Sox9 positive regions (red), respectively. In a *Scx*^*Cre*/*Cre*^ KI mouse, Sox9 expression was markedly decreased in the developing patella and entheseal cartilage (Fig. 9b,h), compared with that in a *Scx*^*Cre*/+^ KI mouse (Fig. 9a,g). Sox9 positive progenitors in the developing deltoid tuberosity of a *Scx*^*Cre*/+^ KI mouse (Fig. 9g) were absent in a *Scx*^*Cre*/*Cre*^ KI mouse (Fig. 9h). Similarly, phosphorylation of Smad1/5 and Smad3 observed in a *Scx*^*Cre*/+^ KI mouse (Fig. 9c,e,i,k) was markedly decreased especially in the prospective patella and deltoid tuberosity of a *Scx*^*Cre*/*Cre*^ KI mouse (Fig. 9d,f,j,l).

### Gene expression of *Col14a1, Decorin, Mohawk, Tnmd* and *Egr1* in *Scx^Cre/Cre^* KI mice

**Figure 10:**
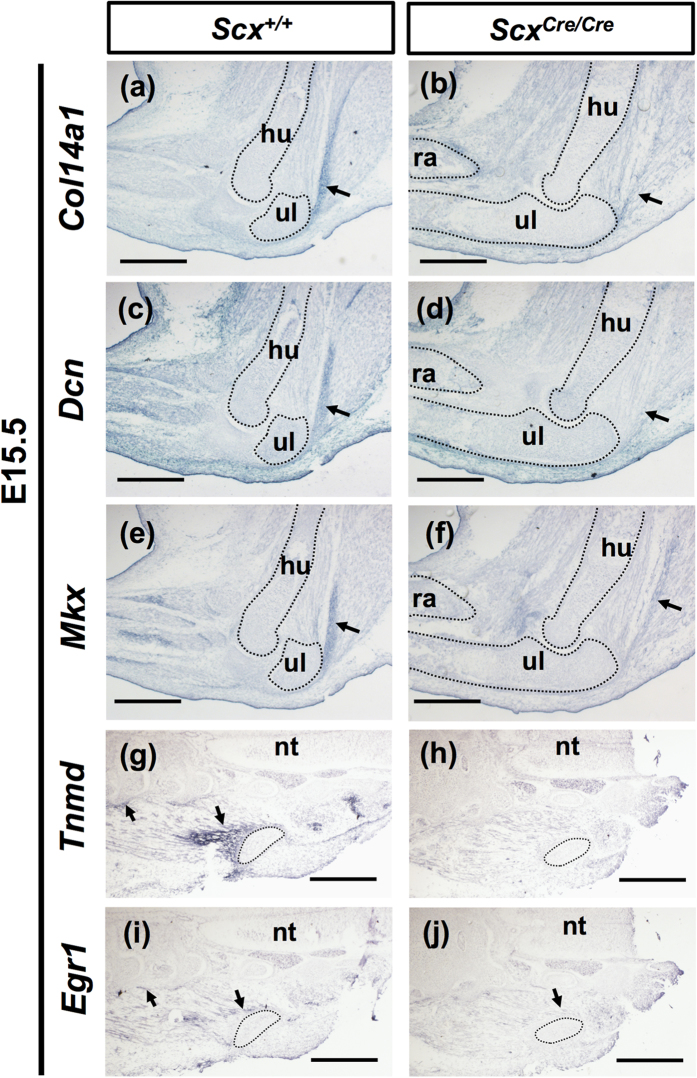
Expression of genes related to tendon formation in *Scx*^*Cre*/*Cre*^ KI mice. (**a**–**j**) *In situ* hybridisation of *Col14a1* (**a**,**b**), *Dcn* (**c**,**d**), *Mkx* (**e**,**f**), *Tnmd* (**g**,**h**), and *Egr1* (**i**,**j**) on sagittal sections of forelimbs (**a**–**f**) and frontal sections of trunks (**g**–**j**) prepared from *Scx*^+/+^ (**a**,**c**,**e**,**g**,**i**) and *Scx*^*Cre*/*Cre*^ (**b**,**d**,**f**,**h**,**j**) KI embryos at E15.5. Arrows in (**a**–**f**) indicate the triceps brachii tendon. Arrows in (**g**,**i**,**j**) indicate *Tnmd* (**g**) and *Egr1* (**i**,**j**) positive tendons. Dotted lines in (**a**–**j**) enclose humerus, radius, ulna, and pelvic bone. hu, humerus; nt, neural tube; ra, radius; ul, ulna. Scale bars, 500 μm.

In addition, we investigated the expression of other tendon-associated genes such as *Col14a1, Decorin (Dcn*), *Mohawk (Mkx*), *Tnmd*, and *Early growth response 1 (Egr1*) in *Scx*^*Cre*/*Cre*^ KI embryos at E15.5. Col14a1 is a member of the fibril-associated collagens with interrupted triple helices (FACIT) collagen family^31^. Dcn is a small leucine-rich proteoglycan involved in regulating collagen fibrillogenesis^32^ and Mkx is a family member of atypical homeobox genes that are expressed in developing tendons^33^,^34^. Egr1 is reported to be a transcription factor regulating the expression of *Col1a1* in tendon development^35^. In a *Scx*^*Cre*/*Cre*^ KI mouse, *Col14a1* expression was almost absent in the developing triceps brachii tendons (Fig. 10a,b). In a wild type mouse, we found expression of *Dcn* in the developing tendon, dermis, and soft connective tissues (Fig. 10c), which are consistent with previous findings^32^. In *Scx*^*Cre*/*Cre*^ KI embryos, we found that *Dcn* expression was almost absent in triceps brachii tendons (Fig. 10d) and also found generally weak expression of *Mkx* (Fig. 10e,f). Around the pelvic bone and lumbar at E15.5, *Tnmd* expressing tendons were also positive for *Egr1* (Fig. 10g,i). In *Scx*^*Cre*/*Cre*^ KI embryos, expression of *Tnmd* and *Egr1* was lost and decreased in theses tendons, respectively (Fig. 10h,j). These findings suggest that loss of *Scx* affects the expression of genes related to tendon maturation.

## Discussion

We successfully generated *Scx*^*Cre*^ KI mice that can be used as genetic tools to reveal *in vivo* function of *Scx*-expressing tissue domains integrating the musculoskeletal components. In *Scx*^*Cre*^ KI mice, *Cre* expression was driven by *Scx* promoter and endogenous *Scx* was inactivated. Cre-mediated recombination efficiency of *Scx*^*Cre*^ KI mice reflected the intensity and duration of endogenous *Scx* expression. Cre-mediated excision was mainly observed in domains with persistent *Scx* expression, such as tendons, ligaments, and annulus fibrosus of intervertebral discs. In homozygous *Scx*^*Cre*/*Cre*^ KI mice, Tnmd, a marker of mature tenocytes and ligamentocytes, was almost absent. In addition, other tendon markers such as *Col14a1, Dcn, Mkx*, and *Egr1* were downregulated in homozygotes. Moreover, regardless of the short duration of endogenous *Scx* expression, defective maturation was observed in *Scx*-expressing domains that are involved in integration of the musculoskeletal components.

*Scx* is localised in the intron 3 region of *Bop1* gene on chromosome 15. *Bop1* and *Scx* are a pair of bidirectional overlapping coding genes related to cellular proliferation and differentiation, respectively^7^,^22^. The first reported *Scx* knockout failed to form mesoderm and died during early stages of embryogenesis^24^, probably because the neomycin phosphotransferase gene linked to phosphoglycerate kinase promoter (*PGK-Neo*) cassette affected the expression of *Bop1*. Later, Murchison *et al*. reported generation and characterisation of a conditional allele in the *Scx* locus with *FRT-Neo* cassette, by flanking the first exon, which includes most of the coding region, with *loxP* site^7^. As was the case with homozygotes of *Scx* knockout with a *Neo* allele, we too failed to obtain viable *Scx*^*CreNeo*^ homozygous pups, but we successfully established a new line of *Scx*^*Cre*^ KI mice by mating a *Scx*^*CreNeo*^ heterozygote with a flippase-expressing *FLPe* mouse. Homozygous *Scx*^*Cre*/*Cre*^ KI mice with tendon defects were viable and similar to *Scx* knockout mice generated by Murchison *et al*.^7^, suggesting that the replacement of most of the exon 1 by *Cre* does not cause embryonic lethality, unlike as observed on retention of *PGK-Neo* cassette in this genomic region.

Cre-mediated recombination in a *Scx*^*Cre*/+^; *Rosa-tdTomato* mouse was less efficient than that in a *ScxCre-L* or a *ScxCre-H* transgenic mouse mated with a *Rosa-tdTomato* reporter mouse. Under the control of the endogenous *Scx* promoter/enhancer in KI mice, *Cre* expression gradually increased to reach levels sufficient for recombination, thus enabling Cre-mediated *tdTomato* expression not in the *Scx*^+^ progenitor cell population but in the differentiated cell populations of tendons, ligaments, the annulus fibrosus of the intervertebral discs, and patella cartilage. More tdTomato positive cells were observed in *Scx*^*Cre/Cre*^; *Rosa-tdTomato* mice than *Scx*^*Cre*/+^; *Rosa-tdTomato*, suggesting that the number of cells expressing *Cre* above the threshold increases in homozygotes due to a gene dosage effect. In the *Cre-loxP* system, recombination occurs only when Cre protein is accumulated above the recombination threshold. Mosaic and later Cre-mediated recombination is considered to be observed due to heterogeneity in the endogenous *Scx* mRNA levels and a narrow time window of *Scx* expression.

We previously reported that *Scx* positively regulates the expression of *Tnmd* in tenocytes both *in vivo* and *in vitro*^6^. *Tnmd* expression in *Scx*-positive cells in the periodontal ligament is also upregulated by lentiviral overexpression of *Scx* and downregulated by knockdown of endogenous *Scx*^36^. In this study, immunostaining of *Scx*^*Cre*/*Cre*^ homozygotes with anti-Tnmd antibody revealed that Scx was necessary for the expression of *Tnmd* in most parts of the developing tendinous and ligamentous tissues. As previously reported, Tnmd acts as a positive regulator of postnatal tendon growth, maturation of collagen fibres, and cellular adhesion in the periodontal ligament^17^. Loss of *Tnmd* resulted in abated tenocyte proliferation leading to reduced tenocyte density, greater variation in collagen fibril diameters, and increase in maximal fibril diameters^16^. Thus, ablation of *Tnmd* expression in tendons, ligaments, and annulus fibrosus of intervertebral discs of homozygous KI mice suggests defective phenotypes in these tissues, other than the previously reported defects in force-transmitting and intermuscular tendons.

In the spinal column, an intervertebral disc lies between adjacent vertebrae and acts as a shock absorber. Intervertebral discs consist of inner and outer annulus fibrosus of sclerotome origin and notochord-derived nucleus pulposus. The inner annulus fibrosus has cartilaginous matrix associated with Col2 fibres, whereas the outer annulus fibrosus has thick multiple layers of dense connective tissue containing predominantly Col1. We previously reported that Scx^+^/Sox9^+^ progenitor population gives rise to both inner and outer annulus fibrosus and that *Sox9* in this population is indispensable for the formation of inner annulus fibrosus^2^. In this study, we found that Tnmd expression in the outer annulus fibrosus was missing in *Scx*^*Cre*/*Cre*^ KI neonates. Thus, we demonstrated, for the first time, that *Scx* is necessary for the maturation of outer annulus fibrosus.

*Scx* is also transiently expressed in *Sox9*-expressing entheseal and sesamoid cartilage during early stages of chondrogenic differentiation, as previously reported^1^,^3^,^37^. This is consistent with our previous findings from lineage analysis using *ScxCre* transgenic mice that entheseal and patella chondroprogenitors were positive for *Scx*^3^. In the *Scx*-expressing domain, mice lacking *Sox9* lose the cartilaginous domain that contributes to the establishment of tendon/ligament attachment sites^2^,^4^. In this study, we found that expression of Sox9 in Scx^+^/Sox9^+^ chondroprogenitors is markedly decreased. Even though *Scx* expression in Scx^+^/Sox9^+^ chondroprogenitors is only transient, loss of *Scx* caused defective formation of cartilaginous elements arising from the Scx^+^/Sox9^+^ progenitor population. Moreover, activation of BMP and TGF-β signalling pathways in these cartilaginous elements was diminished as evidenced by decreased phosphorylation of Smad1/5 and Smad3. These findings indicate that transient expression of *Scx* in chondroprogenitors is functionally important in the process of entheseal cartilage formation.

Muscle contractions *in utero* generate mechanical forces that are essential for normal embryonic development, through modulation of cell signalling and gene expression. As reported previously, *Scx* is the mechanical stress responsive gene that is upregulated in the periodontal ligament in response to tensile force and maintains its fibrogenic state by inhibiting mineralisation^36^. Conversely, removal of tensile force in the tendon results in a decrease in Scx expression^38^. The severe defective phenotypes, initially in force-transmitting and intermuscular tendons and later in ligaments of *Scx*^*Cre*/*Cre*^ KI mice lacking Scx, raised the possibility that *Scx* expression in response to mechanical force during embryonic and postnatal growth might be required for the proper development and structural maintenance of these tissues. Further investigation of target genes regulated by tensile force-responsive Scx may reveal how Scx participates in the regulation of formation and maintenance of tendons and ligaments during development and growth, in the presence or absence of mechanical stress. Such studies are now underway.

## Materials and Methods

### Animals and embryos

Mice were purchased from Japan SLC (Shizuoka, Japan) or from Shimizu Laboratory Supplies (Kyoto, Japan). The generation and establishment of *ScxGFP* transgenic strains have been reported previously^3^. For analysis of Cre activity, *Rosa-CAG-LSL-tdTomato (Rosa-tdTomato*) mice obtained from Jackson laboratory were used^26^. Cre-mediated recombination was monitored by red fluorescence of tdTomato expression driven by CAG promoter. All animal experimental protocols were approved by the Animal Care Committee of the Institute for Frontier Life and Medical Sciences, Kyoto University and the Committee of Animal Experimentation, Hiroshima University, and conformed to institutional guidelines for the study of vertebrates. All methods were carried out in accordance with relevant guidelines and regulations.

### Generation of *Scx* ^*Cre*^ KI mouse strain

The *Scx*^*CreNeo*^ targeting vector was designed to insert the coding region of *Cre*, along with a translational stop codon followed by FRT-flanked *PGK*-*Neo* cassette, in place of the ATG codon of *Scx* coding region, with simultaneous deletion of most of the *Scx* exon 1 (Fig. 1a). For construction of the targeting vector, a fused fragment, containing 706 bp *Scx* genomic region and *Cre (706ScxCre*)^3^, was cut with *Bgl*II from pBluescript SK (+) (Stratagene). *706ScxCre* was inserted into the *Bam*HI site of *pPE7neoW-F2LF* with *PGK-Neo* cassette flanked by two FRT sites, so that the *PGK* promoter was oriented in the opposite direction to the *Scx* promoter. The 486 bp *Scx* genomic fragment downstream of *Bgl*II site in the first exon and first intron was amplified by PCR using a forward primer with *Hin*dIII and *Bam*HI sites (Scx-547F: 5′-AAGCTTGGATCCAGATCTGCACCTTCTGCCTCAG-3′) and a reverse primer with *Hin*dIII and *Not*I sites (Scx-intR2: 5′-AAGCTTGCGGCCGCGGAGGGGTAGTGGCAC-3′). The *Bam*HI site within Scx-547F was introduced for genotyping using Southern blot analysis. The *Hin*dIII-digested amplified fragment was inserted into the *Hin*dIII site of *pPE7neoW-F2LF. PGK-Neo* cassette with homology arms was then used for modification of bacterial artificial chromosome clone (RP23-415D19, CHORI) by using Red/ET recombination system (Gene Bridges) to generate *ScxBAC-CreNeo*, in which *Cre* was introduced in-frame with ATG of *Scx* exon 1 and replaced with a 548 bp region. The approximately 0.5 kb homology arms, homologous to extreme 5′ and 3′ ends of the genomic region to be retrieved from *ScxBAC-CreNeo*, were amplified by PCR. The 5′ homology arm was amplified with forward primer (F1: 5′-AAGCTTCGTTTGCCATCCAGGTCATAACCC-3′) and reverse primer (R1: 5′-ATCGATCCTTGTAAACTACCCACTTGTACCTAAG-3′). The 3′ homology arm was amplified with forward primer (F2: 5′-ATCGATGACAGGCCAGGCCGTGTTCCTGTG-3′) and reverse primer (R2: 5′-CTCGAGCCACATGGCCACTTCACCTCCTTCCTAC-3′). The resultant amplified 5′ and 3′ homology arms were subcloned into *Hind*III-*Cla*I and *Cla*I-*Xho*I sites of *pMCS-DTA*, respectively. For generation of the *Scx*^*CreNeo*^ targeting vector, the modified genomic region with 1.7 kb short and 6.3 kb long arms was retrieved from *ScxBAC-CreNeo* and transferred to *pMCS-DTA*. The PacI-linearised *Scx*^*CreNeo*^ targeting vector was electroporated into KY1.1 ES cell line (F1 hybrid of C57BL/6J and 129S6/SvEvTac)^23^. Total 151 ES cell clones were obtained by positive and negative selection and then screened by PCR using forward primer (F3: 5′-GGATCCTCCTGGGCCCACAGGTCATAG-3′) and reverse primer (Cre-R1: 5′-CTTGCGAACCTCATCACTCGTTGCATCG-3′) (Fig. 1a). We obtained one correctly targeted ES clone that was microinjected into the blastocysts of BDF1 hybrid to generate chimeric mice.

### Genotyping

Mice were genotyped using tail tip genomic DNA by PCR or Southern blot analysis. The wild type *Scx* allele was detected with forward primer (F4: 5′-CTGGTGGGTGAGGCCTGTGGC-3′) and reverse primer (R3: 5′-GAGTCTGTGTCCCAAGGTATG-3′). The *Scx*^*Cre*^ allele was detected with forward primer (Cre-F1: 5′-TCCAATTTACTGACCGTACACCAA-3′) and reverse primer (Cre-R2: 5′-CCTGATCCTGGCAATTTCGGCTA-3′; Fig. 1b). For Southern blot analysis, 10 μg genomic DNA was digested with *Hin*dIII and the resulting DNA fragments were separated via 0.7% agarose gel electrophoresis. After transfer onto Nytran membranes with Turboblotter system (Schleicher and Schuell), hybridisation was performed using probes labelled with [α-^32^P]- deoxycytidine triphosphate (dCTP) (PerkinElmer).

### Northern blot analysis

Total RNA was extracted from wild type and *Scx*^*Cre*/+^ KI embryos at E12.5. Fifteen micrograms of the total RNA was denatured with 6% formaldehyde, fractionated using 1% agarose gel electrophoresis, and transferred onto Nytran membranes with Turboblotter system. A specific cDNA probe for *Scx* was labelled with [α-^32^P]- dCTP. Hybridisations were performed as previously described^6^,^21^.

### Histology

*Scx*^*Cre*/+^; *Rosa-tdTomato* and *Scx*^*Cre*/*Cre*^; *Rosa-tdTomato* neonates were fixed in 4% paraformaldehyde dissolved in phosphate-buffered saline (PFA/PBS) at 4 °C for 3 h, immersed in 20% sucrose/PBS, frozen, and cryosectioned to a thickness of 8 μm. After washing with PBS, nuclei were counterstained with 4′,6-diamidino-2-phenylindole (DAPI; Sigma). Cre-mediated tdTomato expression was detected with a fluorescent microscope. For histological evaluation of knee joint during postnatal growth, paraffin sections prepared from 2-week-old *Scx*^*Cre*/+^ and *Scx*^*Cre*/*Cre*^ KI mice were rehydrated and stained with haematoxylin and eosin. The images were captured under the Leica DMRXA microscope equipped with the Leica DC500 camera (Leica Microsystems).

### Immunostaining

*Scx*^*Cre*/+^KI, *Scx*^*Cre*/*Cre*^ KI, *Scx*^*Cre*/+^; *Rosa-tdTomato*, and *Scx*^*Cre*/*Cre*^; *Rosa-tdTomato* neonates were immersed in 20% sucrose/PBS, frozen, and cryosectioned to a thickness of 8 μm. Sections were fixed in ice-cold acetone prior to double immunostaining of Chmd/Col1, Chmd/Tnmd, and myosin heavy chain (MHC)/Tnmd. For double immunostaining of Chmd and Sox9, p-Smad1/5, or p-Smad3, *Scx*^*Cre*/+^ and *Scx*^*Cre*/*Cre*^ KI embryos were fixed in 4% PFA/PBS at 4 °C for 3 h, immersed in 20% sucrose/PBS, frozen, and cryosectioned to a thickness of 6 μm. For undecalcified sections, 4-week-old *Scx*^+/+^ and *Scx*^*Cre*/*Cre*^ KI mice were anesthetised to perfuse with 4% PFA/PBS containing 20% sucrose. The legs were dissected, fixed in 4% PFA/PBS containing 20% sucrose for 3 h, embedded in SCEM (Leica Microsystems), frozen, and cryosectioned to a thickness of 4 μm, according to Kawamoto’s film method^39^. The sections were decalcified with 0.25 M EDTA/PBS for 60 min and processed for immunostaining. After washing with PBS, the sections were incubated with 2% skim milk in PBS for 20 min and then incubated overnight at 4 °C with primary antibodies diluted with 2% skim milk in PBS. The sections were incubated with appropriate secondary antibodies conjugated with Alexa Fluor 488 or 594 (Life Technologies) and washed again with PBS. The primary antibodies used were anti-Tnmd (diluted 1:1000)^2^,^12^, anti-Col1 (1:500; Rockland), anti-Chmd (diluted 1:500; Cosmo Bio), anti-Col2 (diluted 1:500; Rockland), anti-Ocn (1:800; TaKaRa), anti-sclerostin (1:500; R&D), anti-MHC (1:400; sigma), anti-Sox9 (1:800; Millipore), anti-p-Smad1/5 (1:50; Cell Signaling), and anti-p-Smad3 (1:250; Rockland). Nuclei were counterstained with DAPI. Images were captured using either a Leica DMRXA microscope equipped with a Leica DC500 camera (Leica Microsystems) or an OLYMPUS IX70 microscope equipped with an OLYMPUS DP80 camera (OLYMPUS).

### *In situ* hybridisation

Antisense RNA probes for each gene were transcribed from linearised plasmids with a digoxygenin (DIG) RNA labelling kit (Roche) as previously described^40^. For RNA probes, the cDNAs for *Col1a1, Dcn, Mkx*, and *Egr1* were amplified by RT-PCR using the following primers (mCol1a1_F1: 5′-CCCTCAAGAGCCTGAGTCAGC-3′), (mCol1a1_R1: 5′-GTATTCGATGACTGTCTTGCCC-3′), (mDcn_F1: 5′-TGGTGCAGTGTTCTGATCTGG-3′), (mDcn_R1: 5′-GACTCACAGCCGAGTAGGAAGC-3′), (mfcMkx_F1: 5′-GCGGCCGCCCCCAATCATGAACACCATCG-3′),(mfcMkx_R1: 5′-GCGGCCGCTAGAAGCGCTGCACCAGTGGCA-3′), (mEgr1_F2: 5′-TCTCTTCTTACCCATCCCCAGTGC-3′), and (mEgr1_R2; 5′-TACTGAAATGATTTATCCAATACCATG-3′). RNA probes of *Scx*, and *Tnmd* were described previously^2^,^10^. Mouse *Col14a1* cDNA was also amplified using primers described previously^7^. For *in situ* hybridisation on frozen sections, mouse embryos were fixed with 4% PFA/PBS for 3 h, and treated in 20% sucrose before embedding in Tissue-Tek OCT compound (Sakura Finetek). Seven-micron-thick frozen sections of embedded embryos were prepared and postfixed with 4% PFA/PBS for 10 minutes at room temperature and carbethoxylated twice in 0.1% DEPC/PBS. Sections were treated in 5 × SSC, and hybridisation was performed at 58 °C with DIG-labelled antisense RNA probes. To detect DIG-labelled RNA probes, immunological detection was performed using an anti-DIG antibody conjugated with alkaline phosphatase (Anti-DIG-AP Fab fragment, Roche) and BM purple (Roche). Images were captured using an IX70 microscope equipped with a DP80 camera (OLYMPUS).

### Skeletal preparation

Mouse neonates were dehydrated with ethanol. After removing skin and soft tissues, the neonates were stained with 0.015% Alcian blue 8GX (Sigma) and cleared with 2% potassium hydroxide. The neonates were then stained with 0.05% Alizarin Red S (Wako) in 1% KOH and cleared with 1% KOH.

## Additional Information

**How to cite this article:** Yoshimoto, Y. *et al*. *Scleraxis* is required for maturation of tissue domains for proper integration of the musculoskeletal system. *Sci. Rep.* **7**, 45010; doi: 10.1038/srep45010 (2017).

**Publisher's note:** Springer Nature remains neutral with regard to jurisdictional claims in published maps and institutional affiliations.
